# 970. Medical Student Engagement with the COVID-19 Pandemic Is Increased Through Participation in a Preclinical Elective

**DOI:** 10.1093/ofid/ofab466.1165

**Published:** 2021-12-04

**Authors:** Kaelyn C Cummins, Katherine Naeger, Prathit A Kulkarni

**Affiliations:** 1 Baylor College of Medicine, Houston, Texas; 2 Baylor College of Medicine / Michael E. DeBakey VA Medical Center, Houston, TX

## Abstract

**Background:**

The medical field’s response to the Coronavirus Disease 2019 (COVID-19) pandemic required a multifaceted approach. Medical students were often excluded from the initial phases of pandemic response, resulting in feelings of disengagement. This study aimed to determine if offering educational experiences on current events related to the COVID-19 pandemic could increase medical students’ understanding of, and engagement with, the pandemic.

**Methods:**

In Fall 2020, an elective course reviewing several aspects of the COVID-19 response was implemented. Preclinical medical students attended a discussion-based seminar series given by expert faculty on a variety of topics including pathophysiology, vaccine development, telemedicine, and others. Upon course completion, students were asked to complete a survey quantifying their understanding of the overall COVID-19 response, understanding of various individual facets of the response, and feelings of personal engagement on a Likert scale from 1-5, with 5 representing the most understanding or engagement. The differences in pre-course and post-course mean scores were compared using a Wilcoxon matched-pairs signed rank test for each question.

**Results:**

A total of 65 students completed the course; 35 (54%) students filled out the final course survey. Results showed significant improvement in students’ perceived holistic understanding of the pandemic response (2.67 pre-course vs. 4.36 post-course; p < 0.001), and their feelings of personal engagement (3.06 pre-course vs. 4.33 post-course; p < 0.001). Students also reported significantly increased feelings of understanding for each individual facet of the pandemic response reviewed during the course (8 questions total, all p-values < 0.001).

**Conclusion:**

Preclinical medical student participation in a course reviewing COVID-19 pandemic response significantly increased feelings of engagement with and understanding of the medical field’s response to the pandemic. Students showed improved understanding of each aspect of the pandemic response that was covered in the elective. Therefore, it appears that seminar- and discussion-based electives can be a useful tool for fostering preclinical student engagement in current events in medicine.

**Disclosures:**

**Prathit A. Kulkarni, M.D.**, **Vessel Health, Inc.** (Grant/Research Support)

